# A Case of Disseminated Cryptococcal Infection and Concurrent Lung Tuberculosis in a Patient under Steroid Therapy for Interstitial Pneumonia

**DOI:** 10.1155/2015/358926

**Published:** 2015-06-01

**Authors:** Aoi Kuroda, Sadatomo Tasaka, Kazuma Yagi, Takao Mochimaru, Tetsuo Tani, Ho Namkoong, Kyuto Tanaka, Yusuke Suzuki, Mami Hatano, Naoki Hasegawa, Yasunori Okada, Tomoko Betsuyaku

**Affiliations:** ^1^Division of Pulmonary Medicine, Department of Internal Medicine, Keio University School of Medicine, Japan; ^2^Division of Pulmonary Medicine, Department of Internal Medicine, Kawasaki Municipal Hospital, Japan; ^3^Department of Pathology, Keio University School of Medicine, Japan; ^4^Center for Infectious Diseases and Infection Control, Keio University School of Medicine, Japan

## Abstract

Both disseminated cryptococcal infection and tuberculosis occur in hosts with impaired cell-mediated immunity, but there have been few reports about the concurrent infections in patients without human immunodeficiency virus infection. A 64-year-old man, who had been taking corticosteroids for interstitial pneumonia, was diagnosed with disseminated cryptococcal infection. While the patient was receiving anticryptococcal therapy, pulmonary tuberculosis also emerged. The patient developed acute exacerbation of interstitial pneumonia and passed away. Based on the patient's clinical course, serial computed tomography images, and autopsy results, we believe that the preceding several months of corticosteroid treatment might have contributed to these coinfections in the lungs already vulnerable due to underlying fibrosis.

## 1. Introduction

Disseminated cryptococcal infection and tuberculosis are both known to occur in immunocompromised hosts, such as patients with human immunodeficiency virus (HIV) infection and diabetes mellitus and patients taking immunosuppressants [1]. However, there are a limited number of reports regarding* Cryptococcus neoformans* and* Mycobacterium tuberculosis* coinfection [2–13] (Table 1). Here, we report a case of disseminated cryptococcal infection and concurrent pulmonary tuberculosis in a patient under prolonged corticosteroid treatment who experienced fatal acute exacerbation of previously diagnosed interstitial pneumonia.

## 2. Case Presentation

In April 2012, a 64-year-old man previously diagnosed with interstitial pneumonia presented to a local hospital, complaining of increasing dyspnea over the preceding 3 months. Because exacerbation of his interstitial pneumonia was considered, the patient was hospitalized and 60 mg/day oral prednisolone (PSL) was administered. As his dyspnea improved, the PSL dose was tapered by 5 mg every 4 weeks following discharge.

On September 2, almost three months after discharge, the patient suddenly developed a fever around 38°C and felt intermittent pain in his left lower leg. At that time, he was taking 25 mg of PSL daily. Although he developed steroid diabetes, his HbA1c levels were controlled within the normal range. When he presented to the emergency room of our hospital, his left lower leg had a localized reddish appearance and was slightly edematous without snowball crepitation. Although his consciousness was slightly impaired (Glasgow Coma Scale; E4V4M6), a cerebrospinal fluid (CSF) test without India ink staining was normal. Blood culture tests turned out to be negative. He was hospitalized with a diagnosis of acute bacterial cellulitis.

After admission, the patient received intravenous meropenem. His fever subsided and his mental status returned to normal within days. On day 23 after admission, his leg was no longer swollen or reddish, but the localized pain had not completely disappeared. In order to rule out malignancy and collagen vascular diseases, a skin biopsy of his left ankle was done, and it revealed* Cryptococcus neoformans* infection. We performed a second lumbar puncture, and India ink staining of the CSF also revealed* Cryptococcus* (Table 2). Since a latex agglutination test showed that both his serum and sputum were also positive for* Cryptococcus* antigen, we diagnosed the patient with disseminated cryptococcal infection.

**Figure 1:**
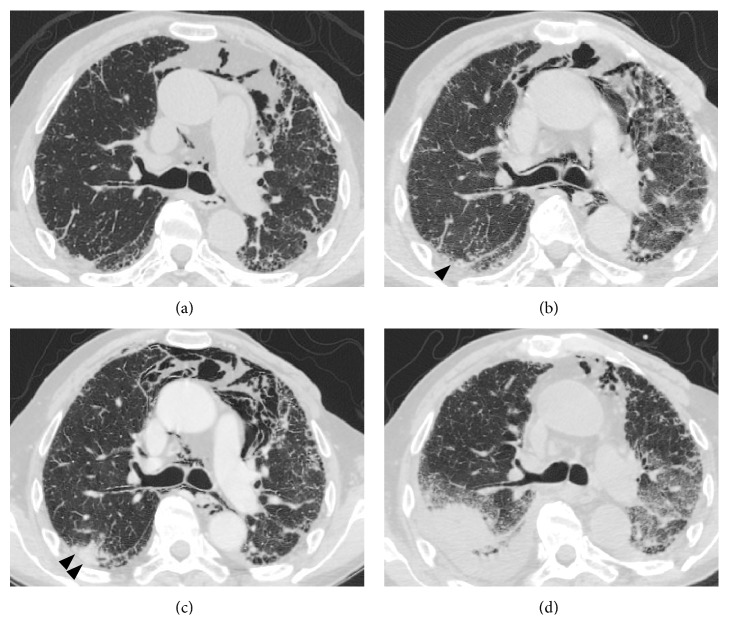
Serial changes in chest computed tomography findings. (a) Computed tomography (CT) scan taken 3 months before admission, showing reticular opacities and small cystic airspaces predominantly in subpleural regions. Mediastinal emphysema is also visible. (b) CT scan taken at admission, showing minimal mediastinal emphysema without major signs of pneumonia. Small centrilobular nodules are visible in the right upper lobe (arrowhead). (c) CT scan taken on the 20th day of hospitalization, showing consolidation in the right upper lobe (arrowheads). (d) CT scan taken on the 77th day of hospitalization, showing increased consolidation size with emergence of diffuse ground-glass opacities in both lungs.

A chest computed tomography (CT) scan taken on day 20 showed that a consolidation in the right upper lung lobe had increased from approximately 25 mm to approximately 40 mm in size (Figures 1(b) and 1(c)); this observation was considered to be asymptomatic focal pneumonitis due to* C. neoformans*. On October 4 (day 30), we initiated antifungal treatment with 250 mg/day (3.6 mg/kg/day) of intravenous liposomal amphotericin B (L-AMB) and 7,000 mg/day (100 mg/kg/day) of oral 5-fluorocytosine (5-FC). After 2 weeks of antifungal treatment, a follow-up CSF culture was negative, suggesting that the treatment was effective. Leukocytosis and serum CRP levels gradually decreased, and his leg pain disappeared. No meningeal signs were observed throughout the treatment course.

**Figure 2:**
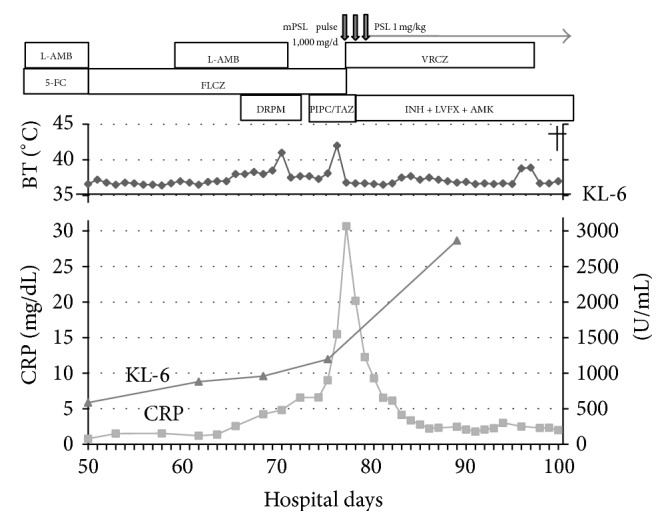
Clinical course. PSL: prednisolone, mPSL: methylprednisolone, L-AMB: liposomal amphotericin B, 5-FC: 5-fluorocytosine, FLCZ: fluconazole, VRCZ: voriconazole, DRPM: doripenem, PIPC/TAZ: piperacillin/tazobactam, INH: isoniazid, LVFX: levofloxacin, AMK: amikacin, BT: body temperature, KL-6: sialylated carbohydrate antigen KL-6, and CRP: C-reactive protein.

Nevertheless, the patient developed acute kidney failure and watery diarrhea possibly due to the L-AMB and 5-FC treatments, respectively. On day 50, we discontinued L-AMB and 5-FC and started the patient on 400 mg/day of oral fluconazole (FLCZ). When we confirmed that the side effects had abated, we added L-AMB again (Figure 2). Despite 4 weeks of cryptococcosis induction therapy, a CT scan on day 71 showed bilateral pleural effusions and further growth of the consolidation in the right upper lung lobe. However, based on repeated negative CSF culture results, we continued maintenance antifungal treatment using FLCZ.

On November 20 (day 77), the patient had a fever over 40°C and severe oxygen desaturation. A CT scan revealed diffuse ground-glass opacities in both lungs, which suggested acute exacerbation of interstitial pneumonia (Figure 1(d)). We started pulse intravenous steroid therapy with methylprednisolone (mPSL) 1,000 mg/day for three days, followed by intravenous PSL 1 mg/kg/day. However, this only halted the exacerbation, and the patient did not improve. On day 78, sputum PCR tests on both day 72 and day 78 turned out to be positive for* M. tuberculosis* (Table 2). Since the patient had respiratory failure and had difficulty taking oral medication consistently, we decided to treat him with isoniazid (300 mg/day), amikacin (250 mg/day), and levofloxacin (750 mg/every other day) intravenously. After 2 weeks of antituberculosis treatment, acid-fast staining tests of his sputum were negative. However, his respiratory failure did not improve, and multiple organ failure developed gradually. He passed away on the 104th day of admission, and his body was autopsied.

The autopsy results were as follows. The lungs had developed significant fibrosis with honeycombing, particularly in the lower lobes. There were areas with diffuse alveolar damage characterized by dense infiltration of inflammatory cells and hyaline membranes. These findings were consistent with the clinical diagnosis of exacerbation of interstitial pneumonia.* Cryptococcus* was found mainly in the pleural membranes as well as in several lung lobes and the prostate gland; however, it was undetectable in the skin or CSF.* M. tuberculosis* was histologically observable as a caseous necrosis in the right upper lung lobe, where CT scans had revealed a 4 cm size consolidation (Figure 1(c), arrowheads). Furthermore, a slight amount of cytomegalovirus (CMV) was detectable throughout the bilateral lungs and seminal vesicles.

Because the patient had no known factors for immunosuppression other than prolonged steroid treatment, serum levels of autoantibodies against interferon-*γ* (IFN-*γ*) and granulocyte macrophage colony-stimulating factor (GM-CSF) were measured. Neither anti-IFN-*γ* nor anti-GM-CSF antibodies were detected in the serum.

## 3. Discussion

We present a case of disseminated cryptococcal infection in a non-HIV patient who had received systemic corticosteroids for several months prior to admission. Consolidation in the right upper lung lobe, which was first considered to be a primary focus of cryptococcal infection, grew in spite of antifungal treatment and was revealed to be tuberculosis during pathological examination. Although combination therapies targeting both cryptococcosis and tuberculosis seemed effective, the patient developed fatal multiple organ failure following acute exacerbation of interstitial pneumonia. The present case indicates that multiple pathogens should be considered for opportunistic infections in patients undergoing prolonged steroid therapy.

Serial changes in the CT appearance and laboratory tests as well as the autopsy findings indicated that this patient might have developed pulmonary infection within only three months due to three different pathogens:* C. neoformans*,* M. tuberculosis*, and CMV. Since tuberculosis screening by acid-fast staining of his sputum and interferon-*γ* releasing assay results were not definitive until day 78 (Table 2), development of tuberculosis was considered unlikely. However, CT images on the day of admission (Figure 1(a)) showed small nodules with centrilobular distribution in the right upper lung lobe, where the consolidation developed later in the disease course. Retrospectively, the small nodules on the CT images could have represented early radiological signs of tuberculosis. In addition, CMV pneumonia, which was not treated in the disease course, might have contributed to the fatal acute exacerbation of interstitial pneumonia. An intensive examination such as bronchoscopy could have detected* M. tuberculosis* and CMV at an earlier stage.

Although cryptococcosis and tuberculosis are both known to occur in immunocompromised hosts, there have been limited reports regarding concurrent cryptococcosis and tuberculosis infections [2–13]. Huang et al. and Kakeya et al. reported 8 and 3 cases of concurrent infection of* C. neoformans* and* M. tuberculosis*, respectively [8, 13]. However, as explained by the authors in the former report, the diagnoses of the two pathogens did not always accurately account for disease pathogenesis: for example, they diagnosed cryptococcosis by only elevated cryptococcal antigen titer in clinical specimens. As summarized in Table 1, more than half of the reported cases had no underlying disease [2, 3, 6–11], which suggests that concurrent development of cryptococcosis and tuberculosis is rare but possible even in immunocompetent, non-HIV patients.

The present case also involved steroid-induced diabetes, which might have contributed to the development of the infection. We considered that, in addition to the prolonged corticosteroid treatment for interstitial pneumonia, there might have been a host factor that increased susceptibility to infection. It has been reported that neutralizing anti-IFN-*γ* autoantibodies are detectable in a large number of non-HIV Asian adults with multiple opportunistic infections [13], but none were detected in our patient. During the disease course, the patient's serum immunoglobulin G (IgG) levels decreased from 1,268 to 583 mg/dL, while his CD4 level remained at 458/*μ*L (day 30). Impaired humoral immunity, which might be associated with prolonged administration of systemic corticosteroids, could have been responsible for multiple opportunistic infections in our patient.

In this case, the tuberculosis diagnosis was delayed because a newly developed lung consolidation was presumed to be focal pneumonitis due to* C. neoformans* infection. Moreover, the CMV infection was not diagnosed during his lifetime since CMV antigenemia tests were repeatedly negative (Table 2). We usually consider multiple abnormal shadows within lungs to have the same etiology. However, pulmonary cryptococcal infections and pulmonary tuberculosis are known to produce a variety of patterns of CT findings, such as consolidations, small nodules, and pleural effusion, especially in an immunocompromised host [14, 15]. The present case suggests that prolonged steroid therapy could be associated with multiple opportunistic infections caused by different organisms, notably in patients with underlying lung disease.

## Figures and Tables

**Table 1:** Reported cases of concurrent cryptococcosis and tuberculosis in patients without HIV infection.

	Age/sex	Race	Region	Underlying disease	Pathological lesions (*Cryptococcus*/tuberculosis)	Treatment (*Cryptococcus*/tuberculosis)	Outcome	References
1	61M	NA	United States	NA	CSF/lung	AMPH-B/INH, SM	Recovered	[2]

2	69M	Caucasian	United States	NA	Both were detected in the same nodule in the lung	KCZ/INH, RFP	NA	[3]

3	51M	NA	Spain	Chronic epididymitis	Both were detected in CSF at almost the same time	AMPH-B, 5-FC/EB, INH, RFP, PZA	NA	[4]

4	61F	Caucasian	NA	Waldenstrom's macroglobulinemia	CSF, blood/cerebral tissue	NA/EB, INH, RFP, SM	Lost to follow-up	[5]

5	34F	Asian	Saudi Arabia	NA	L4-5 vertebral abscess/right axillary lymph node	FLCZ/EB, INH, RFP, PZA	Recovered	[6]

6	25F	Asian	Italy	NA	Both were detected in CSF from the same sample	FLCZ, L-AMB/EB, INH, RFP, PZA, SM	NA	[7]

7	62 (on average)	NA	Taiwan	*∗*	*∗*	AMPH-B, 5-FC/EB, INH, RFP, PZA	Two patients died due to either of the two primary infections	[8]

8	18F	Asian	Canada	NA	Mediastinal lymph nodes and CSF/right upper lung lobe	AMPH-B, 5-FC/EB, INH, RFP, PZA	Recovered	[9]

9	65M	Asian	NA	NA	Both were detected in a large endobronchial mass	AMPH-B, ITCZ/NA	Lost to follow-up	[10]

10	58F	Asian	Taiwan	NA	Left upper and right lower lung lobe/neck lymph node	FLCZ/NA	NA	[11]

11	45F	NA	Turkey	SLE	Both were detected in CSF at almost the same time	AMPH-B, FLCZ, L-AMB/EB, INH, RFP, PZA	Discharged with neurological impairment	[12]

12	65F	Asian	Japan	Diabetes	CSF/left upper lung lobe	AMPH-B, FLCZ/EB, INH, RFP, PZA	Died due to aspiration pneumonia	[13]

13	56M	Asian	Japan	Diabetes, liver cirrhosis	Lung/lung (multiple nodules)	NA/INH, RFP, PZA, LVFX	Died due to hepatocellular carcinoma	[13]

14	83F	Asian	Japan	Rheumatoid arthritis, diabetes	Lung (revealed in autopsy)/lung, CSF, urine, and stool	No treatment/EB, INH, RFP, PZA	Died due to respiratory and heart failure	[13]

15	64M	Asian	Japan	Interstitial pneumonia	Skin, CSF, lungs, pleural membranes, prostate gland/right lower lung lobe, and right pleural effusion	FLCZ, 5-FC, L-AMB, VRCZ/INH, LVFX, AMK	Died due to respiratory failure	Present case

NA: not available, SLE: systemic lupus erythematosus, CSF: cerebrospinal fluid, L4-5: the 4th and 5th lumbar vertebrae, AMPH-B: amphotericin B, INH: isoniazid, SM: streptomycin, KCZ: ketoconazole, RFP: rifampicin, 5-FC: 5-fluorocytosine, EB: ethambutol, FLCZ: fluconazole, L-AMB: liposomal amphotericin B, PZA: pyrazinamide, ITCZ: itraconazole, VRCZ: voriconazole, LVFX: levofloxacin, and AMK: amikacin.

^*∗*^The article describes a study involving 12 non-HIV patients with coinfection of *Cryptococcus* and tuberculosis. Among the 12 patients, six had underlying diseases such as diabetes and eight had concurrent infections of the two pathogens in lungs, CSF, and other organs.

**Table:** (a)

		Day 1	Day 30	Day 49	Day 70
CSF	Pressure (cmH_2_O)	13	5	6	10
	Cell numbers/*μ*L (total)	7	12	9	3
	(mono)	5	12	9	3
	* *(poly)	2	0	0	0
	Cryptococcal antigen	N/A	N/A	>512	256
	India ink stain	N/A	**Positive**	N/A	N/A
	Cryptococcal culture	N/A	**Positive**	N/A	N/A

**Table:** (b)

		Day 23	Day 30	Day 38	Day 72	Day 78	Day 91
Blood	QFT	Negative			**Undeterminable**		
	Cryptococcal antigen		>512				>512
	CMV antigenemia	Negative	Negative			Negative	

Sputum	Acid-fast staining	Negative		Negative	Negative	**Positive (1+)**	Negative
	TB culture	Negative		Positive^*∗*^	Positive^*∗*^	**Positive**	Negative
	TB PCR	Negative		Negative	Positive^*∗∗*^	**Positive**	Negative
	Cryptococcal antigen		**Positive**				

Bold parts: the results became positive while the patient was alive.

^*∗*^Positive: the test results turned out to be positive after the patient died.

^*∗∗*^Positive: the test result turned out to be positive on day 78.

QFT: QuantiFERON TB-2G test; NA: not available.
